# I-REFF diagrams: enhancing transparency in systematic review through interactive reference flow diagrams

**DOI:** 10.1186/s13643-023-02420-0

**Published:** 2024-01-17

**Authors:** Vickie R. Walker, Courtney R. Lemeris, Kristen Magnuson, Christopher A. Sibrizzi, Kelly A. Shipkowski, Kyla W. Taylor, Andrew A. Rooney

**Affiliations:** 1 Division of Translational Toxicology (DTT), National Institute of Environmental Health Sciences (NIEHS), National Institutes of Health (NIH), Mail Drop K204, Research Triangle Park, P.O. Box 12233, Durham, NC 27709 USA; 2 ICF, Fairfax, VA USA

**Keywords:** Interactive REFerence Flow (I-REFF) diagram, PRISMA, Literature flow diagrams, Systematic evidence maps, Systematic reviews

## Abstract

Systematic review methods are recognized for their rigor and transparency and are widely adapted to frameworks that cover review types such as systematic reviews, scoping reviews, and systematic evidence maps. Reporting guidelines help promote better systematic review practices and detailed documentation of the review process for different types of health research (e.g., PRISMA—Preferred Reporting Items for Systematic Reviews and Meta-Analyses; CONSORT—Consolidated Standards of Reporting Trials; and STROBE—Strengthening the Reporting of Observational Studies in Epidemiology). Transparency in the systematic review process and reporting of results is one of the key advantages of the methods and particularly important for hazard and risk assessments due to the high level of scrutiny these reviews face from scientific, political, and public communities. Data visualizations are important to clearly convey information from a review by helping readers perceive, understand, and assess the displayed information easily and quickly. The study flow diagram is a required element of a systematic review and maps out the number of included and excluded records identified, and the reasons for exclusion. Static literature flow diagrams help viewers readily understand the general review methodology and summarize the number of records included or excluded at each stage of the review. However, such diagrams can be time-consuming to develop and maintain during a systematic review or scoping review, and they provide limited summary-level information. We explored how the use of online systematic review tools such as DistillerSR coupled with visualization software such as Tableau can efficiently generate an Interactive REFerence Flow (I-REFF) diagram that is linked to the literature screening data, thus requiring minimal preparation, and resulting in a simplified process for updating the diagram. Furthermore, I-REFF diagrams enhance transparency and traceability by not only summarizing the records in the review but also allowing viewers to follow specific records throughout the review process. We present an example I-REFF diagram and discuss recommendations for key interactive elements to include in these diagrams and how this workflow can improve efficiency and result in an accessible and transparent interactive literature flow diagram without advanced programming.

**Supplementary Information:**

The online version contains supplementary material available at 10.1186/s13643-023-02420-0.

## Background

Systematic review methods are rigorous and transparent approaches used to answer research questions by identifying and critically appraising studies, synthesizing the body of evidence, and reporting results using a pre-specified process. These methods were originally developed in the field of evidence-based medicine and have since been adapted for conducting literature-based assessments in numerous fields of study and multiple review types, such as scoping reviews and systematic evidence maps. Increased objectivity and transparency are the key principles that have contributed to the widespread adoption of these methods. Literature flow diagrams are one of the critical reporting elements of frameworks and reporting standards [the PRISMA statement [1]] for conducting these reviews because they efficiently convey the literature search and screening process, document how many studies were excluded and why, and show the extent of the body of evidence that was identified for addressing the review’s specific research question.

Literature flow diagrams have historically been manually generated visual representations of the literature screening results, and while they add transparency in the reporting, this traditionally static format presents a number of limitations. These diagrams can be time-intensive for authors to generate and maintain, especially when developing them manually and when a review extends over a period of years and includes literature search updates. We have found that manually generated diagrams have greater potential to introduce errors into the study counts throughout the literature assessment, requiring additional time and effort from authors to quality control, correct, and maintain clear documentation to verify accurate study counts. These diagrams are limited to high-level information in the form of summary study counts which are not typically linked to the underlying studies. While literature flow diagrams should cite included studies (i.e., those relevant to addressing the research question), it can be difficult to match study counts to the actual underlying studies recorded at any particular step. Consequently, readers often cannot easily identify an individual study and track its specific screening results contributing to a lack of transparency that is contrary to the goals of systematic review methods. Although some recent tools and templates have been developed to aid in the creation of literature flow diagrams and reduce the effort required to draft them (e.g., Haddaway et al. [2]), resulting diagrams that link studies to underlying data (e.g., citation information) require coding, and Shiny app applications allow for manual entry of the information without interactive capabilities. In addition, DistillerSR provides a PRISMA flow diagram that tracks references that move through the various stages of the screening workflow, but does not provide an interactive display of reference information. In our work, we outline an approach that incorporates screening data to develop an interactive reference flow (I-REFF) diagram that is compliant with PRISMA reporting and does not require coding.

## Literature flow diagrams can be better

Software and tools currently used to screen literature and visualize data have led to increased efficiency in screening steps in the past several years, and these tools have the potential to revolutionize the way literature flow diagrams are developed. We propose the Interactive REFerence Flow (I-REFF) approach (described in the supplemental material) to leverage these new and increasingly powerful tools in developing interactive literature flow diagrams that are populated from screening data and linked to underlying screening results. The I-REFF approach can increase efficiency during diagram development, help to minimize the potential for errors, and enhance transparency and accessibility. To demonstrate I-REFF, we converted a standard literature flow diagram to the I-REFF format using an example from the field of toxicology for which we had access to the underlying data. We used the literature flow diagram from the 2020 National Toxicology Program scoping review of Potential Human Health Effects Associated with Exposures to Neonicotinoid Pesticides to create an interactive diagram, linked to the screening results (original, static literature flow diagram: Fig. 1 in Boyd et al. [3]; updated, I-REFF diagram).

An interactive reference flow diagram has several advantages over static literature flow diagrams. Linking screening data to the literature flow diagram allows summary counts to be automatically calculated. Therefore, when a change in the summary counts occurs, for example, following a search and screening update, minimal effort is required to update the literature flow diagram. Furthermore, because the screening results are already linked to elements such as reference citations and URLs, greater detail and interactivity can be achieved without additional effort. These elements make the new flow diagram more transparent and much more informative for readers. While the structure and summary-level information of the visual are unchanged, interactive elements now allow readers to quickly and easily identify studies considered in the review. The I-REFF approach provides both the capability to develop a static format that is normally seen in publications, but also an interactive format via a link in the publication for the reader to interact, and search the references in an evaluation. With details of the review readily available, readers have a greater ability to check and confirm, re-create, or build upon the review.

## Conclusions

It is time to move beyond manually generated, static literature flow diagrams as the standard in systematic review methods. The I-REFF approach for generating interactive literature flow diagrams is applicable across literature screening platforms and can be achieved with several visualization software programs. An intermediate data transformation step may be required to ensure screening data exists in a format or structure that meets the requirements of visualization software. Widely available tools, such as Microsoft Power Query for Excel or a KNIME workflow, can minimize the effort required for data transformation.

The impact and potential for these diagrams extend beyond what we have demonstrated with our example and include the following:

1. *The approach of linking literature screening data to literature flow diagrams sets the stage for true automation in generating these diagrams in the future*. By connecting visualization tools directly to literature screening platforms and databases, literature screening results could be viewed in a literature flow diagram in real time and without a separate data transformation step. This integration of tools would require collaboration with the tool developers.
2. *The enhanced transparency of reporting reference information for every study considered in the review strengthens the merits of the systematic review methods.* Readers can more efficiently examine, replicate, and expand upon a review.

The richness of information conveyed in interactive elements establishes a new way to explore systematic review results. In a single literature flow diagram, authors have the potential to communicate information about individual studies, ranging from study characteristics to study quality to URLs for in-depth data visualizations and more.

### Supplementary Information

**Additional file 1.** (Supplemental) Interactive Reference Flow (I-REFF) Diagrams: Purpose and Process for Developing.

## Data Availability

Not applicable.

## Abbreviations

I-REFF diagram: Interactive reference flow diagram
PRISMA: Preferred Reporting Items for Systematic Reviews and Meta-Analyses
CONSORT: Consolidated Standards of Reporting Trials
STROBE: Strengthening the Reporting of Observational Studies in Epidemiology

## References

[1] Page MJ, McKenzie JE, Bossuyt PM, Boutron I, Hoffmann TC, Mulrow CD (2021). The PRISMA 2020 statement: an updated guideline for reporting systematic reviews. PLos Med.

[2] Haddaway NR, Page MJ, Pritchard CC, McGuinness LA. PRISMA2020: An R package and Shiny app for producing PRISMA 2020-compliant flow diagrams, with interactivity for optimised digital transparency and Open Synthesis. Campbell Syst Rev. 2022;18(2):e1230. 10.1002/cl2.1230. PMID: 36911350; PMCID: PMC8958186. 10.1002/cl2.1230 PMC8958186 36911350

[3] Boyd WA, Boyles AL, Blain RB, Skuce CR, Engstrom AK, Walker VR (2020). NTP research report on the scoping review of potential human health effects associated with exposures to neonicotinoid pesticides.

