# Supplementary material for: I-REFF diagrams: enhancing transparency in systematic review through interactive reference flow diagrams
Source: Syst Rev. 2024 Jan 17;13:33. doi: 10.1186/s13643-023-02420-0 (PMC10792898; doi:10.1186/s13643-023-02420-0)
Supplement: Supplementary file 1 — Additional file 1. (Supplemental) Interactive Reference Flow (I-REFF) Diagrams: Purpose and Process for Developing. [file 13643_2023_2420_MOESM1_ESM.docx]

# *Additional File*

# Interactive Reference Flow (I-REFF) Diagrams: Purpose and Process for Developing

Reference flow diagrams are reporting and visualization tools to transparently document the screening process in a literature review. To demonstrate the features and advantages of an interactive figure [or Interactive REFerence Flow (I-REFF) diagram] it is helpful to understand some fundamentals of **literature screening** (see Table 1) and how these figures are used to document the process. Therefore, the first section of this document outlines the steps of literature screening at a high level, describes the associated reporting considerations for a reference flow diagram, and then highlights advantages of the I-REFF approach. The second section describes the process for developing an I‑REFF diagram. The approach is intended to be generalizable across software platforms typically used for screening, transforming, and visualizing study selection results.

To demonstrate the I-REFF approach, we converted a standard, static reference flow diagram from a published review to the interactive reference flow format. This example is outlined in the third section and describes how the I-REFF approach could be implemented during a project. We selected a National Toxicology Program scoping review because there is easy access to the raw screening data, and the review addressed a sufficiently diverse topic to illustrate multiple features of a reference flow diagram. The links below can be used to compare the static version to an I-REFF diagram and may be useful to reference for illustrating features described in later sections.

- Original [static reference flow diagram](https://public.tableau.com/views/GEN-11InteractivePRISMAExample/Info-Page2?:language=en-US&publish=yes&:display_count=n&:origin=viz_share_link)
- New [I-REFF diagram](https://public.tableau.com/app/profile/ntp.visuals/viz/GEN-11InteractivePRISMAExample/Dashboard1)

Finally, we summarize the key advantages of the I-REFF diagram approach and provide a list of best practices and additional considerations.

Table 1. Glossary of Key Terms

| **Terminology** | **Definition** |
| --- | --- |
| Bibliographic database | This type of database is an organized digital collection of references of published literature (e.g., PubMed, Web of Science). |
| Literature screening | Literature screening is the process of reviewing and assessing eligibility of references (i.e., include or exclude) for a literature review. It typically consists of one or more rounds of title/abstract review followed by one or more rounds full-text review. |
| Screening tool | The software used to conduct literature screening is often called a screening tool. |
| Study characterization or tagging | Study characterization is the process of identifying or documenting meta-data of references in a literature review by study characteristics of interest for sorting, filtering or categorizing studies. This process is typically conducted as part of or in addition to screening references for eligibility. Standard documentation incudes study design features (e.g., evidence stream, experimental model) or reason(s) for exclusion. Study characterization is often considered a “light” version of data extraction where information is gathered with limited details that are used to broadly categorize or sort references. |
| Data extraction | Extraction is the process of retrieving data from unstructured data sources like scientific articles into structured and standardized forms to support data analysis and synthesis. Data extraction usually includes more details (i.e., actual data results) compared to the categorical information documented during study characterization. |
| Unique Reference Identifier | Identifiers are numeric or alphanumeric strings for each reference that are used to identify the reference throughout the project and potentially track it in a workflow that may move in and out of different screening tools. |

## Fundamentals of Screening: Tracking and Reporting Study Eligibility During Screening

Reference flow diagrams are used primarily to track and transparently report study eligibility decisions (i.e., inclusion and exclusion) and can also reflect **study characterization** (see Table 1) labels (e.g., evidence streams or study designs) identified during the literature screening process (Figure 1). The screening process, in turn will reflect the scope and objectives of the review. Ideally, these procedures are described in a published protocol for the review (which is a requirement for systematic reviews (1) and highly suggested for scoping reviews and other literature-review formats).


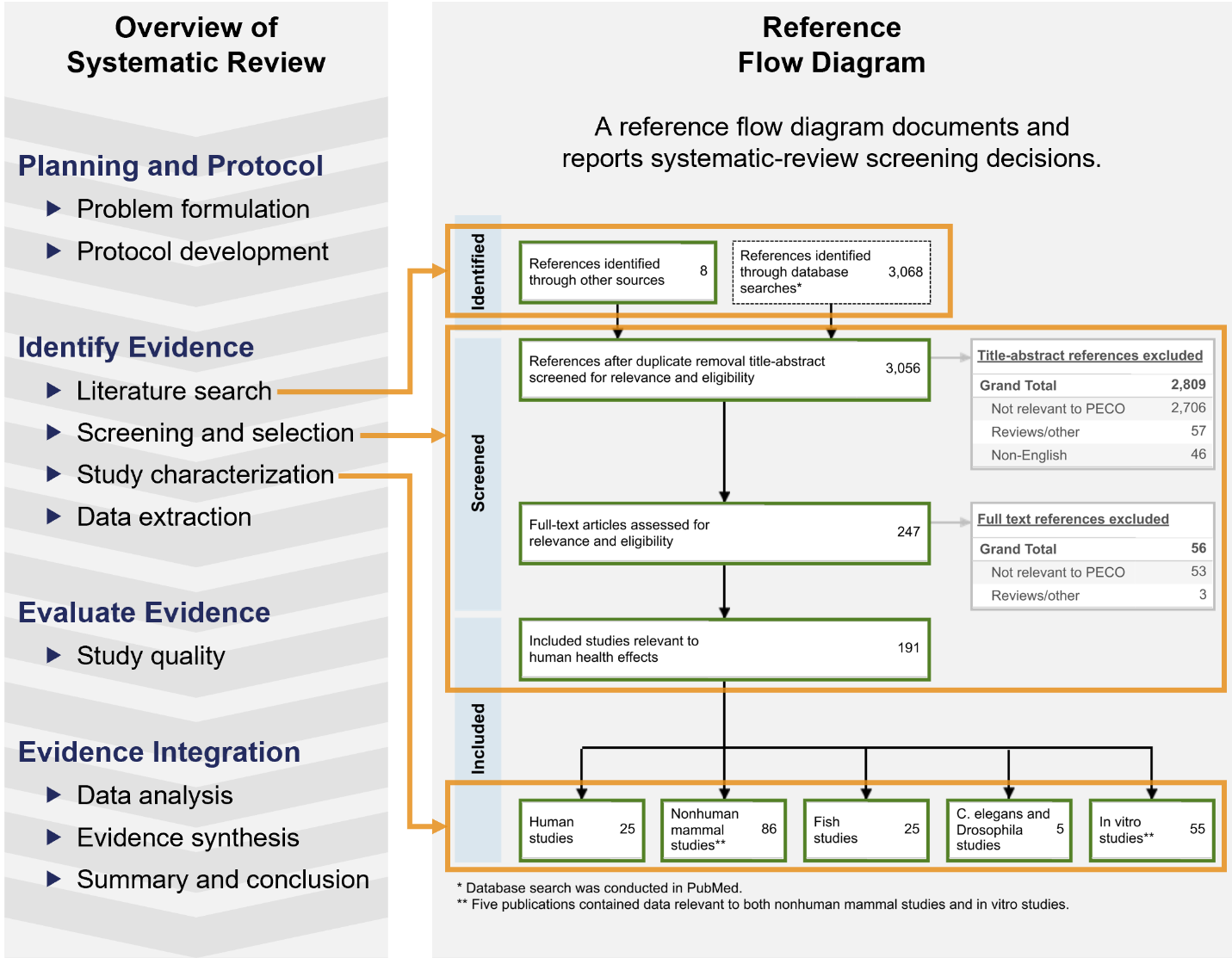


Figure 1. General Systematic Review Process and Its Relationship to Reference Flow Diagrams.

### Title/Abstract Screening

The first step for literature screening is to assess relevancy by reviewing the title and abstract of literature search results. Reference flow diagrams typically document inclusion and exclusion decisions at a high level for this stage, reporting the total number of references considered, included, and excluded. Depending on the objectives of the review, additional details could be documented, such as the reason that studies were excluded. We suggest following reporting guidelines consistent with the goals of the review (e.g., PRISMA for systematic reviews and meta-analyses or MOOSE for meta-analyses of observational studies in epidemiology; (1),(2)) for all of the screening and study categorizations steps.

- ***Input***: Bibliographic reference lists from the literature searches (i.e., literature search results), including database source.
- Action: To document and display screening decisions.
- What to document: Reference flow diagrams should report the number of references retrieved by database (including those identified from other sources such as hand searching of reviews), total number screened at the title/abstract level after removal of duplicates (de-duplication), number excluded at title/abstract level, number unable to be retrieved (if applicable), and number passed on to further steps (i.e., included at title/abstract level). If machine learning approaches are used for screening, the reference flow diagram should distinguish studies excluded by reviewers vs. studies excluded based on machine learning algorithms.
- ***Options and considerations*:** Documentation of reasons for exclusion at title/abstract would represent best practices, although this is not typically done or required. Study types can also be documented, such as reviews that may be identified as supplementary material but excluded from further screening or **data extraction** (see Table 1). Note, the level of detail for reporting in the reference flow diagram or I-REFF diagram should be defined early in the process (i.e., before or early in the literature screening) because these goals will drive the approach for literature screening and study characterization as well as the content, and level of detail in reporting.
- ***Advantages of an I-REFF documentation*:** Typical reference flow diagrams only track the number of references, whereas an I-REFF diagram makes it possible to identify all the individual references included or excluded at the title/abstract level.

### Full-Text Screening

Included references from the title/abstract screening undergo a second screening step to confirm relevance by reviewing the full text of the document. For full-text screening, similar to the title/abstract step, reference inclusion and exclusion decisions are reported. When a reference is excluded at this stage, a reason why the reference was excluded should be documented.

- ***Input:*** Included references from the title/abstract screening step.
- ***Action:*** To document and display screening decisions, including reasons for exclusion of a reference at the full-text level.
- ***What to document:*** Documentation at the full-text level should be the same as title/abstract with the following additions. Studies excluded at the full-text level should provide a reason for exclusion (e.g., not a relevant population), and the methods should specify whether all reasons for exclusion are tracked or just the first one identified. Verify that any study characterization label or category that will inform the final reference flow diagram are represented in the screening form(s).
- ***Options and considerations:*** The reference flow diagram includes a visual representation of the study selection results to the point of presenting the studies that were ultimately identified as included and used for the systematic review. While full-text review is primarily to confirm inclusion and exclusion decisions, in practice this step often includes capturing some metadata (e.g., evidence stream, species, strain, or study design) by which studies can be categorized (see next section on study characterization for additional details).
- ***Advantages of an I-REFF documentation:*** Typical reference flow diagrams only track the number of references and reasons for exclusion at the full-text level; however, an I-REFF diagram can identify all of the individual references included or excluded, as well as reasons for exclusion and through interactive features support searching, sorting or filtering of studies (e.g., by author, title, or exclusion reasons). In addition, a hyperlink to PubMed or DOI record details or data extraction software (e.g., HAWC) could also be provided for a higher level of information.

### Study Characterization

Following full-text review, the included studies are considered for further identification or separation by characteristics of the study. This categorization step identifies and captures limited metadata from references based on the goals of the review. Although we are referring to this step as study characterization, it is important to acknowledge that it represents a process that could be considered part of screening (i.e., where limited metadata are collected during the screening process) or data extraction (i.e., where it represents the first step of capturing detailed study information, including metadata and results). For scoping reviews and systematic evidence mapping, this process is typically conducted as part of screening references for eligibility.

- ***Input:*** Included references from the full-text screening step.
- ***Action:*** Following full-text review, the included studies are considered to further categorize studies beyond simple inclusion which involves tagging studies or capturing limited meta-data from references by study characteristics of interest.
- ***What to document:*** Standard documentation incudes study design features (e.g., evidence stream, study type, experimental model) or reason(s) for exclusion.
- ***Options and considerations***: The level of detail for this step varies significantly between a simple reference flow diagram or typical evidence map. The reference flow diagram includes a visual representation of the study selection results to the point of presenting the studies that were ultimately identified as included and used for the systematic review process, and it relies on clear project objectives and goals. For example, if the goal is to document and display research studies and reviews on the review topic, then these two study types must be included as categories for later visualization.
- ***Advantages of an I-REFF documentation*:** With the I-REFF approach, additional meta-data collected can be included and provided in an interactive, tiered format that allows the user to access a level of detail that they are interested in.

## Developing the Interactive Reference Flow Diagram

Following the screening and study characterization process, developing an I-REFF diagram consists of intermediate steps to ensure data are in a format and structure suitable for the software that will be used to create the interactive visual. The design and format of the final reference flow diagram should clearly demonstrate and communicate study selection results and be easy to interpret and understand. This would apply to both static and interactive reference flow diagrams.

Figure 2 illustrates the steps involved in developing an I-REFF diagram, wherein the screening data are captured, exported, and transformed into a structure suitable for visualization software. The last step is to actually create the diagram. The text below outlines the process for preparing the data for the visualization and identifies helpful screening considerations that will make this process easier.


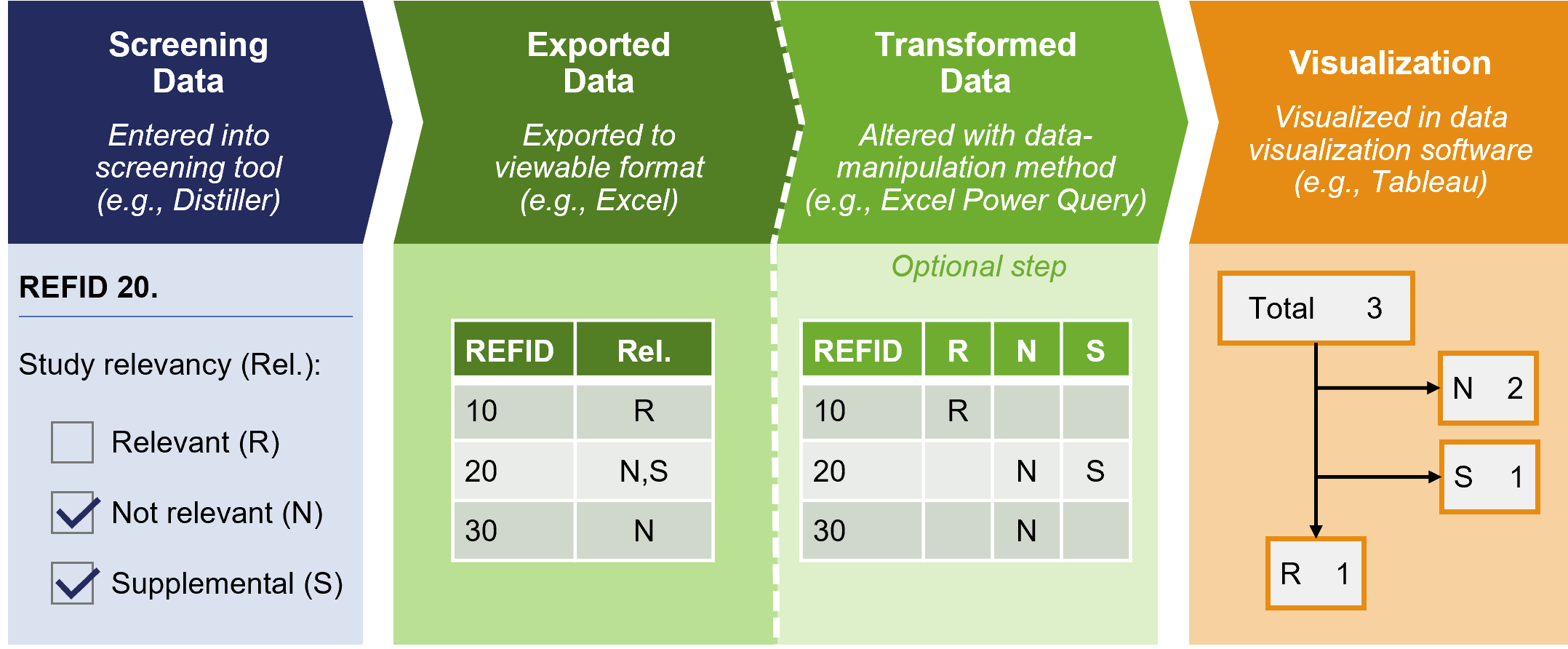


Figure 2. Generalized Approach for Developing I-REFF Diagrams

### Planning Considerations During Screening and Characterization

One of the advantages of an I-REFF diagram is that it not only summarizes screening data, but it is built directly from that data. Consequently, good data management underpins the entire I-REFF approach. Before beginning screening, determine the **screening tool**(s) (see Table 1) that will be used for the project.

- ***Input:*** Project planning during the Problem Formulation or early logistics meetings when planning the literature screening.
- ***Action:*** A **unique reference identifier** (see Table 1) should be used (or assigned if necessary) for each reference that can follow it throughout the project and potentially across software and different screening tools. Verify that all categories or tags that will inform the final reference flow diagram are represented in the screening form(s), as noted in the “What to document” portions of the fundamentals of screening section.
- ***Options and considerations:*** Ideally, project planning includes the identification of study characterization data that are important to display in the reference flow diagram so that all key data are captured during the reference screening and characterization process.

### Export Screening and Characterization Data

Once screening and study characterization are complete, screening data are then exported to a file that is used as the input for creating the I-REFF diagram. Data are either exported in a format that is already properly structured for a direct input to the visualization software or an intermediate step is required to transform the data into the correct structure. If multiple exports are necessary, a transformation step is typically required to stitch the exports together (e.g., if title/abstract review and full-text review are completed in different platforms).

- ***Input:*** Screening data, characterization results and bibliographic reference information.
- ***Action:*** Collect all screening data, study characterization results and reference information into a single spreadsheet file.
- ***Options and considerations:*** Ensure that the export is easily replicable to streamline future updates. For example:
  - Include the same number and/or name for columns in each iteration of an export.
  - Avoid hiding or removing columns of blank data if they might be populated in a later update.
  - To minimize effort, aim to capture all necessary data with the fewest number of data exports possible. Prospective planning to identify metadata of interest during the development of screening and characterization form(s) can help minimize the need for multiple exports and/or data transformation.

### Transform Screening and Characterization Data

If the screening software cannot export the data in an appropriately structured format for the visualization software, a data transformation step is required to manipulate the data into the correct structure. Ideally, this transformation step utilizes tools and methods that can be automated.

- ***Input:*** Spreadsheet file of screening results, study characterization and bibliographic reference information.
- ***Action:*** As needed, restructure (e.g., pivot/unpivot) the rows and columns of data to a structure that aligns with desired reference flow visual data requirements.
- ***Options and considerations*:**
  - In addition to normal data quality assurance/quality controls steps, ensure that data structure and format (i.e., organization of rows and columns) are appropriate for use in the visualization program you intend to use.
  - Use tools to make transforming the data an easily replicable process to minimize effort, if future data updates or changes are expected.
    - For example, Microsoft Power Query for Excel or KNIME can be used to develop a repeatable data process workflow to clean, transform or restructure the data that is then saved and can be reused to refresh the data.
    - For more in-depth manipulation, a script can be written in R (<https://www.r-project.org/>) or Python (<https://www.python.org/>) to run data through more complicated transformation steps to generate properly structured data for visualization.

### Visualize Screening and Characterization Data

The final step is to visualize the data. There are many tools available that can support building and displaying an I-REFF diagram. Ideally, the workflow that takes the data from the screening software to the visualization software is simple and easily repeated to facilitate easy future updates. The visual should represent key steps (e.g., title/abstract and full-text screening recommended in PRISMA or MOOSE) of screening and study characterization. The I-REFF diagram also provides the capability to search, sort, and filter the screening results to view specific studies associated with each of these steps.

- ***Input:*** Data file of screening results, study characterization and bibliographic reference information in a structure consistent with requirements of data visualization software.
- ***Action:*** Load data, build and format I-REFF diagram.
- ***Options* and considerations:**
  - Display study totals on the diagram as the visualization software has ‘counted’ them based on the data entered for each study (i.e., do not hard code totals unless this is necessary).
  - Include a visualization landing page (or “Read me” information) that orients users to the interactive visual and includes instructions for navigation and filtering.
  - Include relevant legends and footnotes that explain visual concepts.
  - Keep the visual design and interface design simple and easy to interpret.
  - Interactive features:
    - Include a reference list that is connected to each other aspect of the diagram, so that specific references can be viewed when the visual is filtered to a specific category total of the diagram.
    - Link diagram totals via filtering, so that if an upstream total is selected, downstream totals are filtered to reflect relevant references.
      - Clearly mark or label any totals that are hard coded and excluded from the interactive filtering functionality.
      - Display counts of total studies associated with each category total and incorporate functionality allowing them to change to accurately reflect applied filters.
      - Use tooltips to provide additional information where necessary (e.g., reference details and/or links).
      - Use clickable buttons to provide any additional information (e.g., detailed instructions) or navigation function (e.g., to send users to a ‘Read me’ page).

## Implementing the I-REFF Approach – Case Study Example (Neonicotinoid Pesticides)

NTP published a *Scoping Review of Potential Human Health Effects Associated with Neonicotinoid Pesticides*, that examines the potential association between exposures to neonicotinoid pesticides and human health effects (3). The data produced in this report were used to create an example I-REFF diagram, the tools and process used to develop the I-REFF diagram are outlined below. This section is intended to demonstrate the process and considerations that could be achieved using a number of different tools. The mention or use of these tools does not indicate any endorsement of these products by NIEHS, nor does it imply fitness for any particular use or purpose of the product.

Specific tools and databases used to support this case study example are listed below.

- A single database, PubMed, was selected to search for potentially relevant literature.
- The PMID number was used as a unique reference identifier to track each reference throughout the review.
- EndNote was used to organize literature search results and facilitate removal of duplicate references.
- Literature search results were screened using DistillerSR®.
- DistillerSR® and Microsoft Excel were used to perform abbreviated data extraction and characterization.
- Microsoft Power Query for Excel was used to transform data in preparation for visualization.
- Tableau® (2022.1.3) was used to create the example I-REFF diagram.

### EXAMPLE Screening (Neonicotinoid Pesticides)

In this section, we describe the screening and characterization process for the neonicotinoid screening.

#### Title/Abstract Screening

At the title and abstract level review, a screening form was developed and applied to determine eligibility based on PECO criteria with the additional exclusion criteria of non-English language publication, and publications that did not contain original data (i.e., review, editorial, and conference abstracts). Screening Question 1 in Table 2 shows question and answer choices for title/abstract screening used in this example.

#### Full-text Screening and Study Characterization (Screening and Study Characterization Combined in One Step)

Full-text screening used a more detailed screening form to confirm PECO eligibility and identify additional study characteristics. The full-text screening form included an inclusion/exclusion question and additional questions to gather study characteristics, including evidence stream, type of exposure and broad health outcome categories, in support of the scoping review objectives. The evidence stream(s) examined in each reference was the only study characteristic reported in the final reference flow diagram in this example; however, the other details could be incorporated into an I-REFF diagram if desired. Screening methods that use machine learning were not used. Screening Questions 2 and 3 in Table 2 display the questions and answer choices used in this example for full-text screening and characterization, respectively.

Table 2. Screening and Characterization Questions, Answers, and Workflow

| **Type of Question** | **Screening Question** | **Application** | **Response Options** | **Screening Result** |
| --- | --- | --- | --- | --- |
| Title/Abstract Screening: Inclusion/Exclusion | 1. Is the study relevant to PECO and evaluates neonicotinoid chemical of interest? | Question #1 is applied to all studies | Yes, Relevant | Include |
|  |  |  | Not Relevant | Exclude |
|  |  |  | Non-English Language | Exclude |
|  |  |  | Review, editorial or conference abstract | Exclude |
| Full-Text Screening: Inclusion/Exclusion | 1. Based on full-text review, is the study relevant to PECO and evaluate neonicotinoid chemicals of interest? | Question #2 is applied to studies included in Question #1 | Yes, Relevant | Include |
|  |  |  | No, Exposure not relevant | Exclude |
|  |  |  | Non-English Language | Exclude |
|  |  |  | Review, editorial or conference abstract | Exclude |
|  |  |  | Not Relevant | Exclude |
| Full-Text Study Characterization:  Evidence Stream | 1. What evidence streams are reported? | Question #3 is applied to studies included in Question #2 | Human | -- |
|  |  |  | Non-Human Mammal | -- |
|  |  |  | Fish | -- |
|  |  |  | C. elegans | -- |
|  |  |  | In Vitro | -- |
|  |  |  | Other | -- |

### EXAMPLE I-REFF Diagram Development (Neonicotinoid Pesticides)

In this section, we describe the process followed to convert the neonicotinoid screening and characterization data into an I-REFF diagram.

#### Exporting Data

As noted above, screening was performed in DistillerSR® and project data were exported from this tool into Microsoft Excel. Exports from DistillerSR® included the following:

- A unique identifier for each reference
- Bibliographic reference information (e.g., citation, abstract)
- All title/abstract screening results
- All full-text screening and characterization results

Table 3 provides an example of the unique reference identifier, reference information, and screening and characterization neonicotinoid data exported from DistillerSR®. An alternative format showing the same data collapsed into fewer columns is presented in

Table 4. For this project, it was possible to include all study data from all screening levels (i.e., title/abstract and full-text) in a single export; however, sometimes it is necessary to split data by level or another criteria if export size becomes a limiting factor.

Table 3. Example Data Exported from DistillerSR® Before Transformation

| **Ref. ID** | **Short Citation** | **Screening Step** | **Title/Abstract Screening Result** | **Full-Text Screening Result** | **Human Evidence** | **Non-Human Mammal Evidence** |
| --- | --- | --- | --- | --- | --- | --- |
| 1 | Abou-Donia 2008 | Title/Abstract | Include |  |  |  |
| 1 | Abou-Donia 2008 | Full Text |  | Include |  | Non-Human |
| 2 | Aprea 2009 | Title/Abstract | Include |  |  |  |
| 2 | Aprea 2009 | Full Text |  | Exclude |  |  |
| 3 | Abbas 2015 | Title/Abstract | Exclude |  |  |  |
| 3 | Abbas 2015 | Full Text |  |  |  |  |
| 4 | Agha 2012 | Title/Abstract | Include |  |  |  |
| 4 | Agha 2012 | Full Text |  | Include | Human |  |

Table 4. Example collapsed data exported from DistillerSR® before transformation

| **Ref. ID** | **Short Citation** | **Title/Abstract Screening Result** | **Full Text Screening Result** | **Evidence Stream** |
| --- | --- | --- | --- | --- |
| 1 | Abou-Donia 2008 | Include | Include | Non-Human |
| 2 | Aprea 2009 | Include | Exclude | - |
| 3 | Abbas 2015 | Exclude | - | - |
| 4 | Agha 2012 | Include | Include | Human |

#### Transforming Data

Data transformation for this example was conducted using Microsoft Power Query for Excel, according to the data formatting needs of Tableau.

Creating an I-REFF diagram in Tableau requires that data be organized and formatted to fit a few key traits:

1. All data for a single reference should appear in a single row of the data table and be labeled with a unique identifier (1 reference = 1 row).
2. A distinct column of the data table should exist for each screening/characterization study total to be shown on the final diagram (1 total = 1 column).
3. Data entered into screening/characterization columns should use a binary labeling system.

Keep in mind that if Tableau is not your visualization software of choice, these traits may not be essential to create a diagram. However, organizing the data in this fashion should work with most tools.

##### 1 Reference = 1 Row

In this instance, the data exported from DistillerSR® screening forms provided data for single studies across multiple rows (Table 3). In order to develop the visuals, these data were transformed from multiple-rows-per-reference to a single-row-per-reference format (Table 5 or Table 6).

##### 1 Category Total = 1 Column

Interactive functionality between category totals in Tableau also requires a distinct column of data for each total shown on the diagram. So if the data export from DistillerSR® had already been collapsed into a single row per reference (Table 4), it would still need to be formatted like Table 5 or Table 6 to create a distinct column for each of the totals shown on the I-REFF diagram (i.e., a column for title/abstract include and a separate column for title/abstract exclude).

##### Binary Labels

Binary labels (e.g., ‘true/false’ in Table 5 and ‘label/blank’ in Table 6) provide the visualization software a mechanism of counting the number of studies that fit the criteria of each column and displaying each total in the final diagram.

Table 5. Example A: Data after Transformation

| **Ref. ID** | **Short Citation** | **Title/ Abstract Include** | **Title/ Abstract Exclude** | **Full Text Include** | **Full Text Exclude** | **Human Evidence** | **Non-Human Mammal Evidence** |
| --- | --- | --- | --- | --- | --- | --- | --- |
| 1 | Abou-Donia 2008 | True | False | True | False | False | True |
| 2 | Aprea 2009 | True | False | False | True | False | False |
| 3 | Abbas 2015 | False | True | False | False | False | False |
| 4 | Agha 2012 | True | False | True | False | True | False |

Table 6. Example B: Data after Transformation

| **Ref. ID** | **Short Citation** | **Title/ Abstract Include** | **Title/ Abstract Exclude** | **Full Text Include** | **Full Text Exclude** | **Human Evidence** | **Non-Human Mammal Evidence** |
| --- | --- | --- | --- | --- | --- | --- | --- |
| 1 | Abou-Donia 2008 | Include |  | Include |  |  | Non-Human |
| 2 | Aprea 2009 | Include |  |  | Exclude |  |  |
| 3 | Abbas 2015 |  | Exclude |  |  |  |  |
| 4 | Agha 2012 | Include |  | Include |  | Human |  |

##### Additional Transformations

It may be necessary to perform additional transformations on the data to prepare for visualization. For example, if title/abstract and full-text screening data had been exported as 2 separate exports from DistillerSR®, a transformation step combining these 2 exports (either in Excel or in Tableau) would have been necessary. This is an example of the crucial nature of a unique identifier for each reference, since this identifier would be the data point used to ‘stitch’ together multiple exports.

#### Visualizing the Data

Once the data were transformed into this cross-tabular format, with one row corresponding to each reference and one column corresponding to each total to be displayed on the final diagram, the data were loaded into Tableau to create the final I-REFF diagram. We followed the general process described below to create the final interactive dashboard.^[[1]](#footnote-2)^

- 1. Loaded data into Tableau.
  2. Created a worksheet for each category total of the final diagram. Included a label and a Tableau-rendered total count of distinct references. See Figure 3.


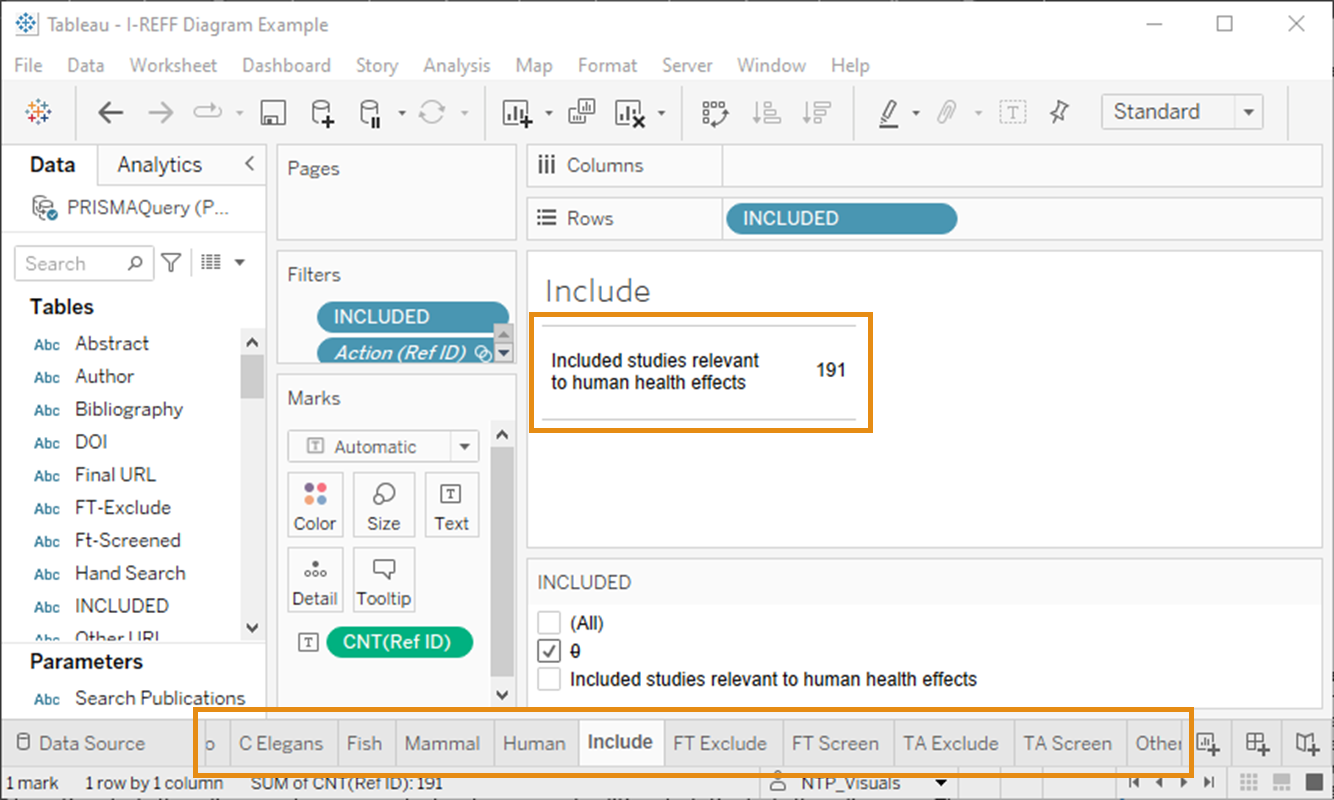


Figure 3. Example of Tableau software showing a worksheet for each total on final diagram.

- 1. Created a worksheet for the reference list. Included short citation labels and icons with additional information in tooltips. Also included a free-text search filter for use on final dashboard. See Figure 4.


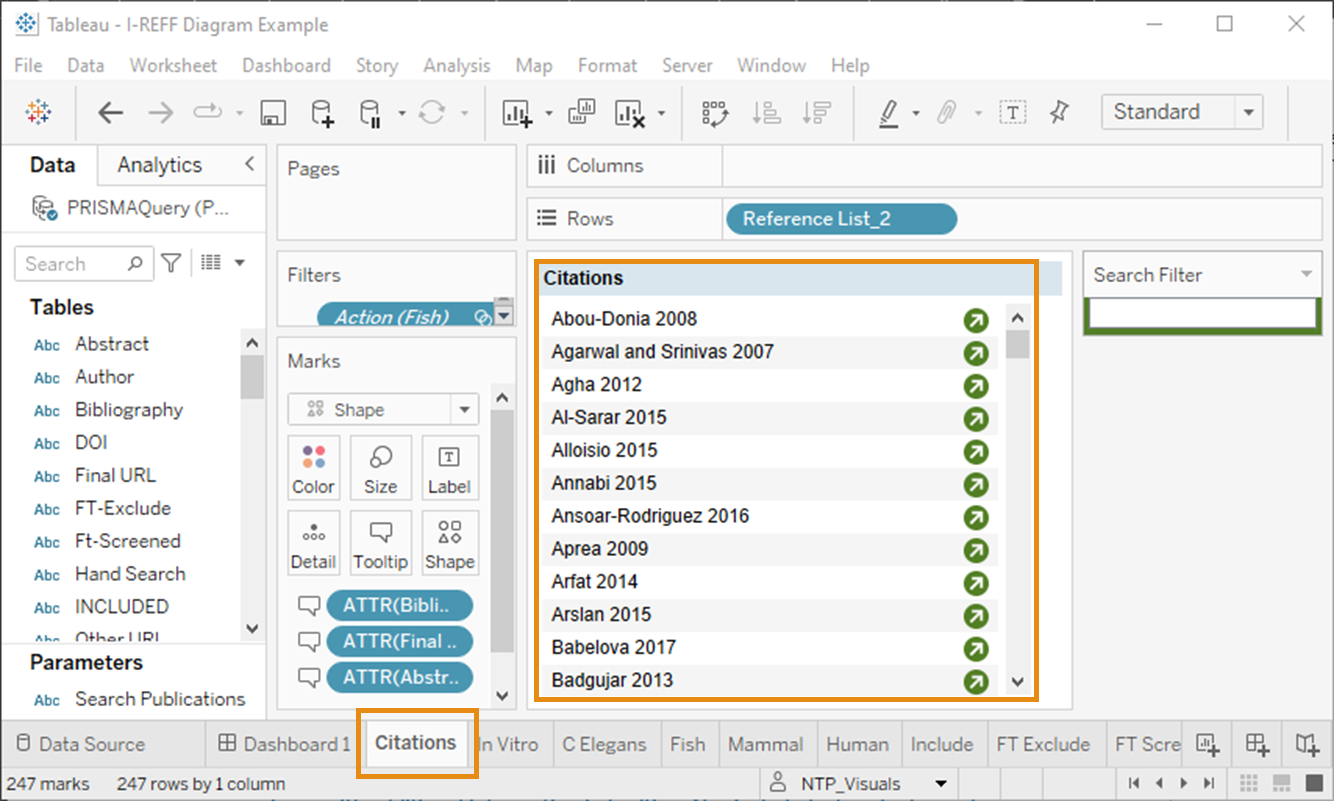


Figure 4. Example of Tableau software showing a worksheet for the reference list on final diagram.

- 1. Developed the flow diagram by placing all worksheets on a single dashboard. Used floating sheets and objects for maximum layout control. Inserted images of arrows where necessary to indicate reference flow. Applied ‘use-as-filter’ functionality to each individual worksheet on the dashboard (Figure 5).


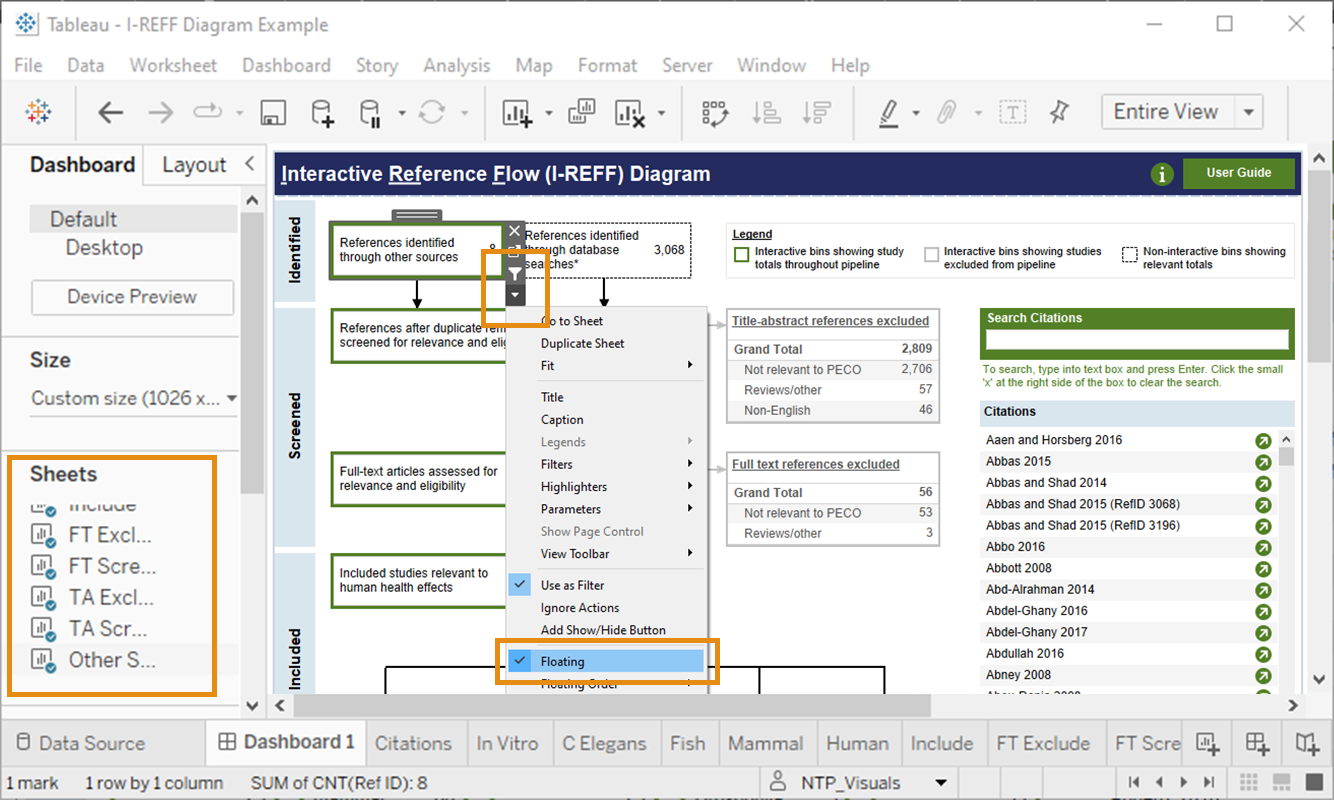
Figure 5. Example of Tableau software showing a dashboard combining worksheets using filter options and floating formatting.

- 1. Added footnotes, legends, and visual instructions where necessary, also using floating objects.
  2. Added navigation and information buttons to take users to and from additional pages of relevant information and instructions.

For comparison purposes, the original static reference flow diagram and the new I-REFF diagram for this example are provided at the following links:

- - Original [static reference flow diagram](https://public.tableau.com/views/GEN-11InteractivePRISMAExample/Info-Page2?:language=en-US&publish=yes&:display_count=n&:origin=viz_share_link)
  - New [I-REFF diagram](https://public.tableau.com/app/profile/ntp.visuals/viz/GEN-11InteractivePRISMAExample/Dashboard1)

## Why Reviewers Should Adopt Interactive Reference Flow Diagrams

### Power of this Approach

I-REFF diagrams have several advantages over traditional, static reference flow diagrams. The fundamental benefit for authors is to improve efficiencies in the process of developing the reference flow diagram and quality assurance over the long-term. The principal advantages for readers are the clarity and richness of information.

#### For Authors:

- - Improves workflow efficiencies by combining data management with development of final visuals into a single process.
    - Especially true for reviews that will undergo data updates.
    - Helps to minimize the potential for miscalculations or reporting errors when calculating summary counts.
  - Makes updates to the reference flow diagram easier.
  - Provides flexibility to include additional reference information while maintaining alignment with standard reporting guidelines.
    - Information can be tiered using tooltips.

#### For Readers:

- - Conveys more information than a static visual, notably comprehensive interactive bibliographic reference lists.
  - More efficient presentation of bibliographic references considered in the review by reducing the need to refer to other files.
  - Creates an interactive experience allowing for new ways to explore and engage with systematic review screening data.
  - Offers a greater opportunity to examine, replicate, and update reviews.

### Future Directions

The steps described above establish an approach for developing I-REFF diagrams using a screening tool and visualization software. As these tools continue to evolve, the goal should be to minimize the steps required to connect underlying screening data with interactive visualization tools and to explore the capabilities of integrating I-REFF diagrams into existing screening platforms.

## Best Practices and Additional Considerations:

### Planning

- Determine the screening tool that best suits the objectives of the literature assessment.
  - Criteria to consider when selecting a screening tool may include, cost, number of users, interoperability with other tools, conflict resolution, quality control capabilities, exporting features, screening form capabilities or advanced screening features (e.g., machine learning, natural language processing that reorder and prioritizes based on relevance).
    - Example platforms include but are not limited to:
      - Covidence (<https://www.covidence.org/>)
      - DistillerSR®(https://www.evidencepartners.com/products/distillersr-systematic-review-software)
      - litstream^TM^ (<https://www.icf.com/technology/litstream>)
      - Microsoft Access or Excel
      - SWIFT Active-Screener (<https://www.sciome.com/swift-activescreener/>)
- Develop screening forms and ensure all categories that will inform a final reference flow diagram are accounted for in the screening phase; you can’t report a category in the reference flow diagram that was not properly captured during the screening phase. Work backwards from the goals of the reference flow diagram and planned products to determine the best structure for screening forms.
  - Consider required groupings of studies (e.g., separating evidence streams such as human vs. animal studies).
  - Consider any additional details downstream from study selection that may be useful to document (e.g., if a study type, such as a case report, was included so it could be tracked but its data were not extracted because it was not comparable to other study types).
- Determine the data visualization software that best suits the objectives of the I-REFF diagram
  - Criteria to consider when selecting visualization software may include, cost, user interface (learning curve), interoperability with other tools, formatting options, search and filter capabilities, and publishing or sharing features.
    - Example tools include but are not limited to:
      - Microsoft Power BI (<https://powerbi.microsoft.com/en-us/>)
      - Qlik Sense (<https://www.qlik.com/us/products/qlik-sense>)
      - RShiny (<https://shiny.rstudio.com/>)
      - Tableau (<https://www.tableau.com/>)

### Screening

- Use fundamental best practices for data management.
  - Ensure references are assigned a unique identifier that follows the reference throughout the project and potentially in and out of different tools.
  - If multiple tools are used for a project, it is strongly suggested to import all data when moving from one tool to the next and not limit the import to just the included references. For example, if the title/abstract screening is completed in one tool and full-text screening completed in a different tool, consider moving all screening results to the full-text platform to have all the screening data in one place.
  - Make any screening update or edit directly in the screening tool. For example, if a study was mistakenly included but should have been excluded for a specific reason, update the data in the screening tool to reflect the correction to avoid a manual correction to the I-REFF diagram downstream

### Visualization

- Identify interactive elements for each stage of study selection that will be incorporated into the I-REFF diagram.
  - At a minimum, all references that were screened should be accessible with 2-way filtering.
    - First, select a category total of studies on the flow diagram (e.g., excluded based on title/abstract review) for a reference list of all studies in that category.
    - Second, select a single study in the bibliographic reference list to see the fate of that study throughout the flow diagram (e.g., at which stage it was excluded and why).
- Include all required reporting elements for a reference flow diagram according to the reporting guidelines selected for the review.
  - Table 7 provides a list of standard reporting elements for all reference flow diagrams, as well as additional elements recommended by reporting guidelines (e.g., PRISMA (1), MOOSE (2)) for all reference flow diagrams.
- Consider additional reporting elements that would enhance the I-REFF diagram.
  - Table 8 provides examples of reporting elements that could be incorporated into an I-REFF diagram. These elements are standard for reviews but not necessarily considered standard for reference flow diagrams (i.e., they are often reported in the write-up of the review).

Table 7. Reporting Elements Typically Included in Reference Flow Diagrams

| **Part of Diagram** | **Reporting Elements** |
| --- | --- |
| **Standard Elements** | |
| *Identification* | References identified through bibliographic database |
|  | References identified through other sources |
|  | References removed before screening (e.g., duplicate references) |
| *Screening* | References screened at the title/abstract stage |
|  | References excluded during title/abstract review   - If automation tools were used, indicate references excluded by a human vs. references excluded by the tool |
|  | References sought for full text retrieval |
|  | Full-text references not retrieved |
|  | References screened at the full-text level |
|  | References excluded during full-text review   - Reason(s) for exclusion during the full-text review |
| *Included* | References included in the review |
| **Recommended Elements** | |
| *Identification* | Source of references identified |
|  | Reason references were removed prior to screening (e.g., duplicate references, references removed by a pre-screen automation tool) |

Table 8. Reporting Elements Not Typically Included in Reference Flow Diagrams That Could Be Incorporated Into I-REFF Diagrams

| **Part of Diagram** | **Reporting Elements** |
| --- | --- |
| **Recommended Elements** | |
| *Reference List* | Bibliographic details and/or hyperlinks for all references considered in the review |
| **Optional Elements** | |
| *Identification* | The date when each literature-search source was last consulted |
|  | Specific details of the source(s) of references identified (e.g., if forward or backward searches were performed, specific bibliographic details of the reference on which the search was applied, the citation index or platform used [e.g., Web of Science], and the date the search was performed) |
| *Included* | Characteristics for each included study |
|  | For meta-analyses, specify references included in the primary analysis or sensitivity analysis |
| *Other Information* | Registration information for the review |
|  | Registration for the review protocol or where the protocol can be accessed |
|  | Literature search strategy details (e.g., search terms used) |
|  | Eligibility criteria |
|  | Screening process details (e.g., number of reviewers, use of automation tools) |

# References:

1. Page MJ, McKenzie JE, Bossuyt PM, Boutron I, Hoffmann TC, Mulrow CD, et al. The PRISMA 2020 statement: An updated guideline for reporting systematic reviews. PLoS Med. 2021;18(3):e1003583.

2. Brooke BS, Schwartz TA, Pawlik TM. MOOSE reporting guidelines for meta-analyses of observational studies. JAMA Surg. 2021;156(8):787-8.

3. Boyd WA, Boyles AL, Blain RB, Skuce CR, Engstrom AK, Walker VR, et al. NTP research report on the scoping review of potential human health effects associated with exposures to neonicotinoid pesticides. Research Triangle Park, NC: National Toxicology Program; 2020. Report No.: 15.

1. This process, while described at a high level that is applicable to several visualization tools, provides illustration using Tableau and occasionally uses language specific to Tableau; general knowledge of Tableau may be useful for the purpose of following the example. [↑](#footnote-ref-2)
